# Unveiling Spin Transition at Single-Particle Level in Levitating Spin Crossover Nanoparticles

**DOI:** 10.1021/acsnano.5c18794

**Published:** 2026-02-03

**Authors:** Elena Pinilla-Cienfuegos, Lucas Mascaró-Burguera, Ramón Torres-Cavanillas, J. Ignacio Echavarría, Alejandro Regueiro, Eugenio Coronado, Javier Hernandez-Rueda

**Affiliations:** † Nanophotonics Technology Center, 16774 Universitat Politècnica de València, Valencia E46022, Spain; ‡ Instituto de Ciencia Molecular, 16781 Universitat de València, Valencia 46980, Spain; § Department of Optics, Faculty of Physics, 16734 University Complutense of Madrid, Plaza de Ciencias 1, 28040 Madrid, Spain

**Keywords:** spin crossover, molecular materials, quadrupole trap, multispectral scattering microscopy, laser-induced spin transition, single-nanoparticle sensing, single-particle photonics

## Abstract

The ability to control and understand phase transitions of individual nanoscale building blocks is key to advancing the next generation of low-power reconfigurable nanophotonic devices. To address this critical challenge, molecular nanoparticles (NPs) exhibiting spin crossover (SCO) phenomenon are trapped by coupling a quadrupole Paul trap to a multispectral polarization-resolved scattering microscope. This contact-free platform simultaneously confines, optically excites, and monitors the spin transition in Fe­(II)–triazole NPs in a pressure-tunable environment, eliminating substrate artifacts. Thus, we demonstrate light-driven manipulation of the spin transition in levitating NPs, enabled by laser heating and free of substrate-induced effects. Using the robust spin bistability near room temperature of our SCO system, we quantify reversible optovolumetric changes of up to 10%, revealing precise switching thresholds at the single-particle level. Independent pressure modulation produces a comparable volume increase, confirming mechanical control over the same bistable transition. These results constitute full real-time control and readout of spin states in levitating SCO NPs, with operating conditions compatible with ultralow-power optical switching, data storage, and nanoscale sensing.

## Graphical abstract

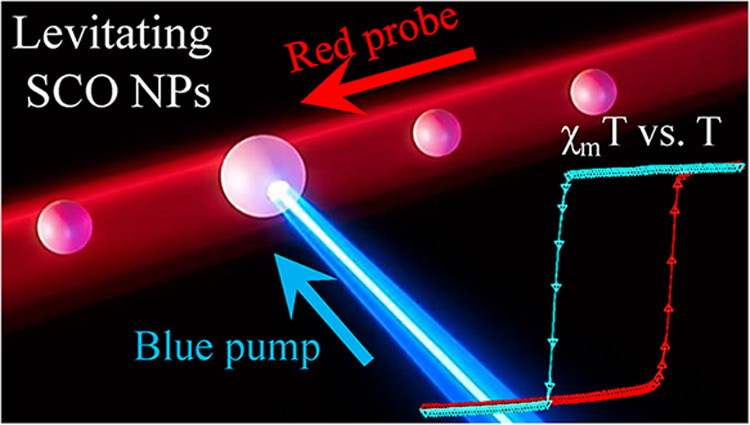

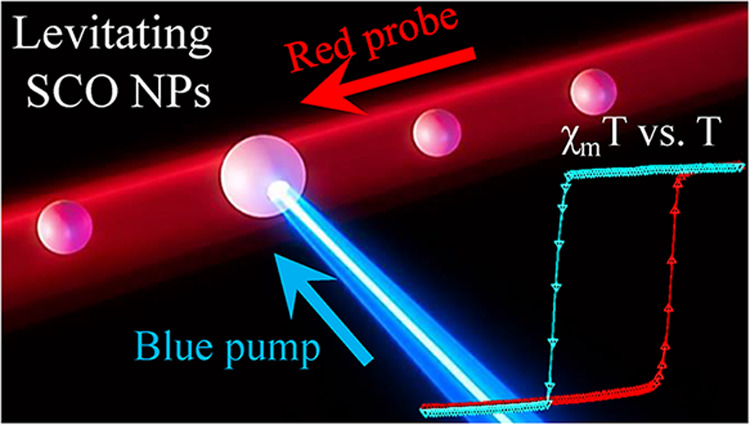

## Introduction

Reconfigurable nanophotonic devices rely on phase-change materials (PCMs) to dynamically control light.^1^ To be suitable for practical implementation in integrated photonic circuits and optoelectronic systems, PCMs must meet key requirements such as high optical contrast, fast and reversible switching, low energy consumption, and scalability.^2^ While conventional PCMs such as chalcogenides and vanadium dioxide satisfy some of these requirements, they are often limited by high switching energy, optical losses, and, in certain cases, poor cycling stability, which hinders their performance and long-term reliability in photonic applications.^3–5^ Molecular PCMs such as spin crossover (SCO) materials stand out as particularly promising candidates, as their properties can be chemically tailored for advanced photonic functionalities.^6–8^ These compounds, generally based on octahedral Fe­(II) coordination complexes, reversibly switch between two electronic configurations, the so-called high-spin (HS) and low-spin (LS) states, in response to external stimuli, such as light irradiation, and temperature or pressure variations.^9–15^ This transition is accompanied by substantial changes in structural (volume), magnetic, optical, electrical, mechanical, and thermal properties that become evident to the naked eye through changes in color.^6,16–18^ Among these changes, the optical response is particularly relevant for photonic integration: spin transition induces a measurable change in the refractive index with a predicted Δ*n* ≈ 0.01–0.1 in the vis–NIR range when transitioning from the HS to the LS. Equally significant is the accompanying reversible volume increase, often of the order of several percent in volume (Δ*V* ≈ 1–10% in typical Fe­(II) complexes), arising from the structural rearrangement between the high- and low-spin configurations.^19^ For these reasons, and since SCO is a molecular phenomenon that can function even at the single-molecule scale, the scientific community has focused in recent years on the development of SCO systems at the nanoscale for their integration into nanodevices.^19–21^ Notably, in many SCO compounds, intermolecular interactions between the molecular metal complexes give rise to a cooperative spin transition with thermal hysteresis, creating a bistable temperature window in which either spin state may be stabilized, a key feature for applications in memory storage and switching technologies.^22–24^

However, manipulation and readout of individual SCO NPs have proven to be a major challenge. Previous studies employing ultrafast electron microscopy to probe mechanically induced or plasmon-assisted spin transitions in supported NPs have provided significant insight, but are inherently limited by substrate-induced effects that can interfere with accurate measurement of their intrinsic properties.^25–27^ This challenge becomes even more pronounced when attempting to integrate individual SCO NPs into functional nanodevices, as illustrated by notable work that involved the integration of ca. 10 nm NPs of the polymeric chain compound [Fe­(Htrz)_2_(trz)­(BF_4_)] (where Trz refers to the triazole ligand) between two gold electrodes.^28^ Interestingly, thermal bistability in transport properties was detected near room temperature, which was associated with the spin transition. However, this device lacked sufficient stability and reproducibility for practical applications. As a consequence, most reported works have relied on researchers using compressed NP powders, embedding particles in polymer matrices, or depositing particles on conducting two-dimensional systems.^29–33^ Some efforts have used soft lithography to position NP assemblies (rather than single NPs) of the aforementioned compound between gold electrodes.^34^ An even more sophisticated procedure exploits the anisotropy of large SCO microrods to trap them between electrodes via dielectrophoresis.^35,36^ Unfortunately, these approaches do not allow precise control of individual SCO nanosystems and typically result in assemblies of nano/microparticles within the device that, although stable, still lack reproducibility and uniformity. In this context, we introduce an approach using a quadrupole Paul trap to isolate SCO NPs in a controlled atmosphere. This platform enables substrate-free, real-time optical control and monitoring of SCO transitions at the single-particle level, with environmental control over pressure, temperature, and laser intensity, while fully eliminating substrate-induced effects. A Paul trap uses an oscillating electric field to confine ions or single NPs in three dimensions.^37–40^ In recent years, quadrupole traps have been shown to be ideal for isolating single NPs, enabling precise spectroscopic studies in a controlled environment.^41–43^ More recently, Paul traps have been used to manipulate single NPs to investigate macroscopic quantum states, leading to insights with great potential for sensing applications with enhanced sensitivity.^44–49^

In this work, we used this procedure to isolate SCO NPs of the amino derivative of the Fe­(II)–triazole compound [Fe­(NH_2_trz)_3_(NO_3_)_2_]. We selected this system for its robustness, sharp hysteretic response, and spin transition centered near room temperature, making it a promising candidate for the design of low-power bistable devices. The trap setup, combined with a multispectral polarization-resolved scattering microscope, allowed us to simultaneously confine, excite, and probe a spin transition at the single-NP level. The transition between spin states was triggered by a tunable external pump laser, with the transition thermally induced by adjusting the laser power. Because the trap operates in a vacuum chamber to ensure stable confinement of NPs, each NP levitates freely in the Paul trap, allowing its full three-dimensional size change to be measured without substrate constraints. Moreover, precise pressure control in the vacuum chamber enables us to actively modulate particle size through pressure-induced dehydration and to monitor volumetric changes in real time. By analyzing the scattering signal and size variations, we were able to identify the spin state of each particle and pinpoint the specific excitation conditions of the laser power at which the spin transition occurs, along with the corresponding size changes induced by vacuum tuning. Finally, we discuss the potential of the investigated phase-change material for developing low-power nanophotonic switches and detectors.

## Results and Discussion

### Ex Situ Characterization of SCO NPs

Fe­(NH_2_trz)_3_(NO_3_)_2_ undergoes a spin transition near room temperature, enabling low-energy activation. Since it contains multiple anions (NO_3_^–^) in its structure, this compound is well suited for the formation of partially charged NPs under applied voltage, which is a key requirement to effectively trap NPs (see Figure S1). NP synthesis was carried out using a reverse micelle protocol, as described elsewhere, which involved mixing two separate microemulsions.^50^ We adapt the experimental conditions to synthesize NPs with sizes that range between 100 and 300 nm (see Experimental Methods section), which was confirmed by dynamic light scattering (DLS, in Figure S2) and transmission electron microscopy (TEM, in Figure 1(a)).

**Figure 1:**
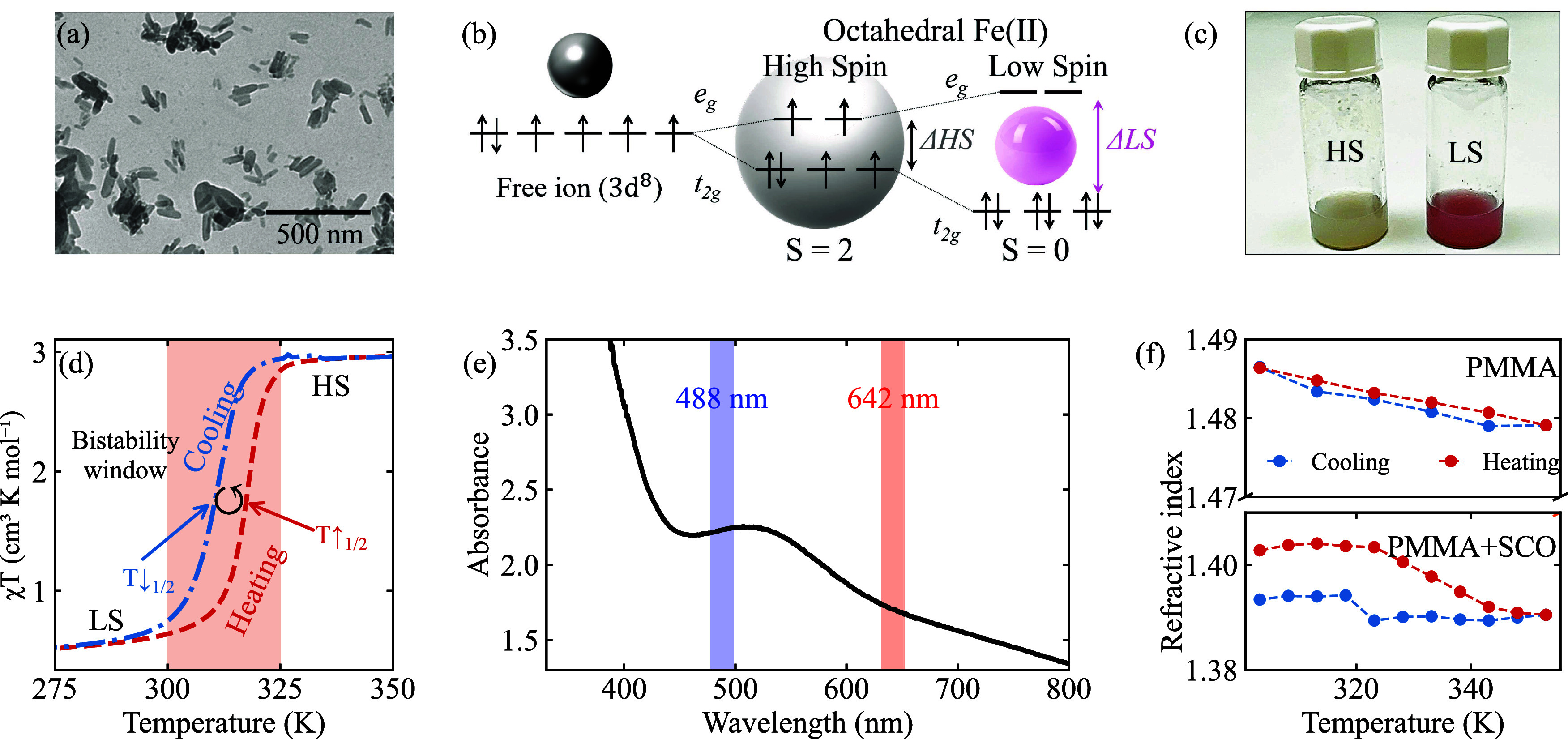
(a) Representative TEM image of Fe­(NH_2_trz)_3_(NO_3_)_2_ particles. (b) Schematic of the electron redistribution between the LS and HS configurations in an octahedral Fe­(II) coordination compound upon different external stimuli (pressure, temperature, and light irradiation). (c) Picture of a bistable SCO/PMMA solution at room temperature with low- (pink, LS) and high-spin (white, HS) states. (d) Heating-and-cooling cycles of *χT* as a function of temperature. (e) Absorption spectrum of NPs redispersed in ethanol. (f) Ellipsometry measurements with heating-and-cooling cycles of the real part of the refractive index for a bare PMMA layer (top graph) and SCO/PMMA film (bottom graph).

We confirmed that the compound was successfully formed using infrared spectroscopy (IR) and X-ray diffraction (XRD) (Figure S3). The energy diagrams presented in Figure 1(b) illustrate the HS and LS configurations for an Fe­(II) complex, which, for the HS state, features four unpaired electrons with *S* = 2 that yield a strongly paramagnetic configuration. Its LS state counterpart has no unpaired electrons and is diamagnetic. This fundamental difference underlies the SCO behavior, which can be triggered by external stimuli such as variations in temperature, pressure, or light intensity, as mentioned in the Introduction section. The distinct number of unpaired electrons in the high- and low-spin states results in a marked change in color and magnetic moment. Figure 1(c) shows a picture of a bistable SCO NP suspension in a poly­(methyl methacrylate) (PMMA) matrix at RT, whose color changes from typical pink at LS to milky white at HS after a heating cycle due to a spin transition. To accurately determine the transition temperature of the synthesized NPs, we investigated their magnetic response by measuring the thermal dependence of χ*T*, in cm^3^ K mol^–1^. Figure 1(d) shows a well-defined thermal spin transition from the diamagnetic LS (*S* = 0) state to the paramagnetic HS (*S* = 2) state at around 320 K (T↑1/2) upon heating. During cooling, the LS state is recovered below 310 K (T↓1/2), demonstrating a reversible spin transition with a bistable window of approximately Δ*T* = 10 K. This hysteretic behavior is consistent across successive thermal cycles.

To optically characterize Fe­(NH_2_trz)_3_(NO_3_)_2_ NPs, we performed ultraviolet–visible (UV–vis) spectroscopy and ellipsometry measurements, as shown in Figure 1(e),(f), respectively. The particles were embedded in PMMA to enable their optical characterization. The room temperature UV–vis absorption spectrum of NPs redispersed in ethanol exhibits the characteristic LS state signature, marked by a broad absorption band centered at 520 nm with a bandwidth of 100 nm. This feature gives the LS state its typical pink color. In contrast, this absorption band disappears in the HS state (see the HS absorption spectrum in Figure S6 and the supporting video), rendering the dispersion milky white, as shown in Figure 1(c). Based on this spectrum, we selected a 488 nm blue laser to trigger the spin transition and a 642 nm red laser for scattering-based size measurements of individual particles. For spectroscopic ellipsometry measurements, the resulting nanocomposite (SCO/PMMA) was spin-coated onto a silicon substrate, producing a thin, optically smooth layer suitable for ellipsometric analysis. This approach allowed us to extract the complex refractive index of the Fe­(NH_2_trz)_3_(NO_3_)_2_ compound and monitor its variation across the spin transition as a function of temperature. The graphs in Figure 1(f) show the real part of the refractive index during heating and cooling cycles for the SCO/PMMA film (bottom panel) and a bare PMMA layer (top panel) at a wavelength of 642 nm. Similarly to χ*T*, the refractive index exhibits thermal hysteresis, while the PMMA film shows a linear behavior. The measurements revealed a refractive index of *n*_LS_ = 1.4028 for the LS state and *n*_HS_ = 1.3905 for the HS state, corresponding to an optical contrast of Δ*n* = 0.0123 across the spin transition (see full ellipsometry characterization in Supporting Information Figure S5).

### Trapping Levitating SCO NPs

To investigate the spin transition at the single-NP level, we make use of a Paul trap embedded in a vacuum chamber and monitor its response to external stimuli using multispectral polarization-resolved scattering microscopy (Figure 2(a)).^41,43^ Continuous-wave probe beams at 642, 785, and 852 nm are combined in a fiber multiplexer, collimated, polarization-controlled, and aligned collinearly with the trap axis. A separate 488 nm CW laser serves as a pump for the optical excitation. Scattered light from the trapped particle is collected perpendicularly to the trap axis by a long-working-distance microscope objective and imaged onto a camera for polarization-resolved scattering measurements (see the Experimental Methods section for details). The inset in the upper right displays a 3D sketch of trapped NPs, highlighting the collinear red probe beams and the perpendicular blue pump beam. This configuration enables simultaneous optical excitation and multiwavelength, polarization-resolved optical detection of single particles while varying environmental parameters such as chamber pressure. We introduce Fe­(NH_2_trz)_3_(NO_3_)_2_ SCO NPs (hereafter SCO NPs) in the trap by using an electrospray system that combines a syringe with an ethanol solution of the NPs coupled to an emitter connected to a high voltage (≈3 kV). The trap entrance is grounded, thus the voltage difference generates a Taylor cone, where particles are separated and propelled toward the trap input port as shown by dark-field and shadowgraphy microscopy in Figure 2(b).^51,52^ The trap consists of four metallic rods diagonally connected to either an AC sinusoidal signal or a constant DC signal, as sketched in Figure 2(c). This configuration generates a quadrupolar potential that traps single NPs along the trap axis, which obey the Mathieu equations of motion. The precise choice of frequency and amplitude of the potential offers the flexibility to trap particles with on-demand sizes; i.e., these traps were originally conceived as mass selectors, where the charge-to-mass ratio plays a crucial role in trapping stability (see more details in Experimental Methods section). Thus, we set the trap setup parameters to trap SCO NPs with a size around 300 nm, which simultaneously provides good trapping stability and a strong scattering signal during experiments (Experimental Methods section). This size is consistent with the size distributions measured by TEM (see Figure 1(a)) and dynamic light scattering (DLS; Figure S2). Our trap setup uses an automated scattering microscope to collect images of isolated SCO NPs illuminated with low-intensity linearly polarized laser light at different polarization angles, as shown in Figure 2(c). Figure 2(d) shows a picture of the Paul trap with an array of SCO NPs levitated in a pressure-controlled environment, which is ideal for studying the spin transition upon laser irradiation and pressure variations.^41,43^ The scattering response of isolated particles provides insight into how they behave under external stimuli, leading to a better understanding of the SCO mechanism at the single-particle level. This method has the potential to reveal strategies to fine-tune the spin transition for specific applications across different environments or nanophotonic platforms.

**Figure 2:**
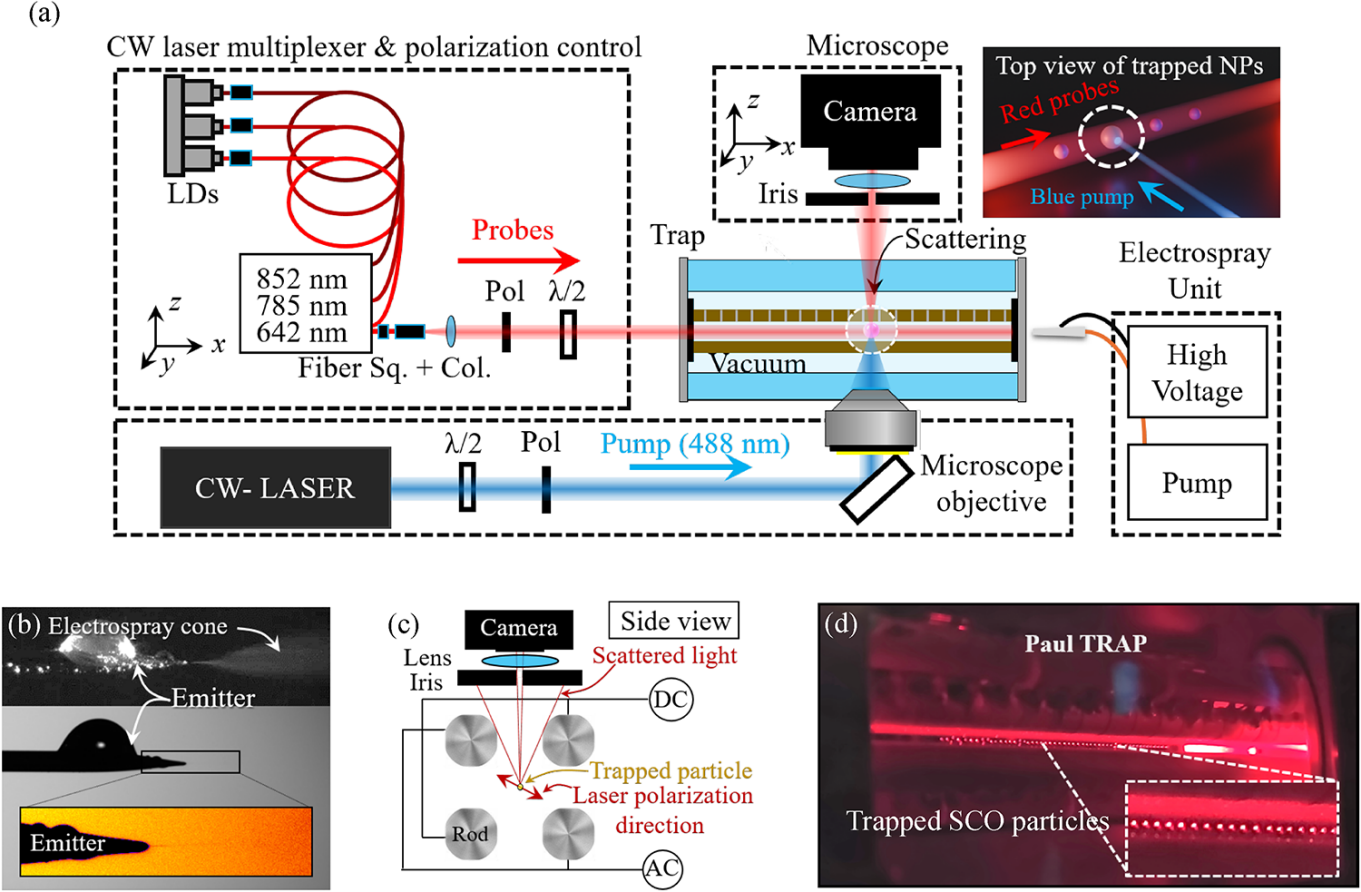
Experimental setup for polarization-resolved scattering measurements on levitated SCO nanoparticles. (a) Schematic of the optical system: three continuous-wave (CW) probe lasers at 642, 785, and 852 nm (LDs) are combined via a fiber multiplexer, collimated, and polarization-controlled by using a half-wave plate (λ/2) and linear polarizer (Pol) before entering the trap along the *x*-axis. A separate CW laser at 488 nm is used as a pump beam when required. The trap is placed inside a vacuum chamber with optical access through a microscope objective, and the scattered light is collected along the *y*-axis and imaged onto a camera. Inset: top-view 3D sketch image of trapped nanoparticles under simultaneous red probe and blue pump illumination. (b) Electrospray source used to inject SCO NPs into the trap. Dark-field and shadowgraphy microscopy images show the emitter and the formation of a Taylor cone. (c) Side-view schematic of the linear Paul trap indicating the positions of the DC end-cap electrodes and AC rods, as well as the polarization direction of the probe laser and the scattered light collection geometry. (d) Photograph of the Paul trap during operation, showing the trapped SCO NPs illuminated by the probe beams.

### Laser-Induced Thermal Control of Reversible Spin Transition in Trapped SCO NPs

In the following, we inspect levitating SCO particles using polarization-resolved scattering microscopy to attain key insights into their optical response and size changes upon a reversible spin transition induced by laser heating from an initial room-temperature state. During each experimental run, trapped SCO NPs are independently illuminated with two perpendicular continuous-wave (CW) laser beams at 488 and 642 nm, as shown in Figure 3(a), which are used to excite the particle and probe its scattering response, respectively. The excitation beam at 488 nm is focused on the NP using a microscope objective (Mitutoyo, 10×, NA = 0.28), while the much less intense collimated probe beam at 642 nm illuminates trapped NPs. Here, the blue laser beam acts primarily as a local heat source, so the observed switching reflects a thermally driven spin crossover.

**Figure 3:**
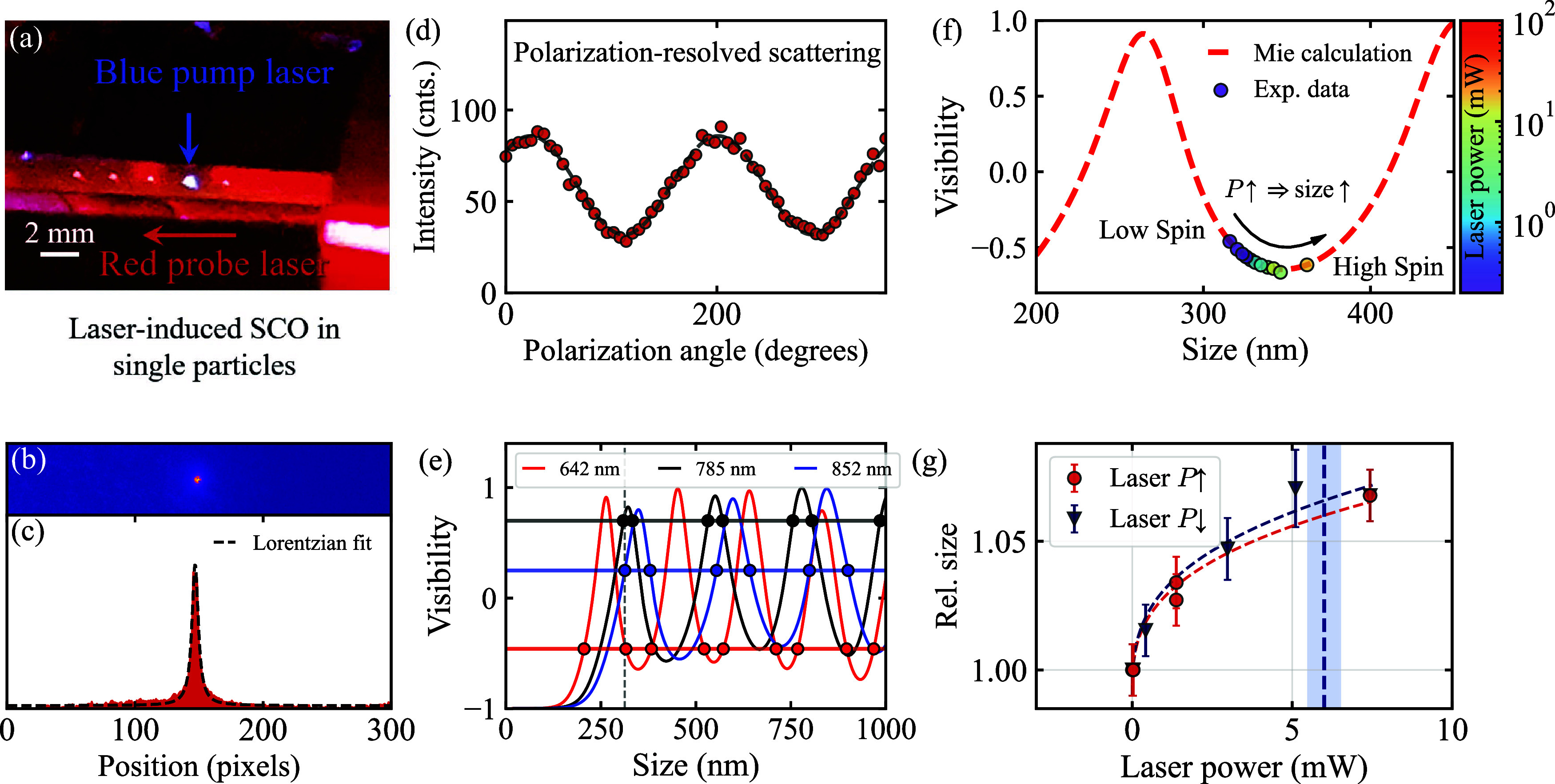
(a) Image of an array of isolated SCO NPs inside the trap, which are illuminated with a collimated red probe laser and excited with a focused blue pump laser. (b) Scattering micrograph and its (c) integrated profile of a trapped NP illuminated with a CW laser at 642 nm. The dashed black line illustrates a fit using a Lorentzian function. (d) Scattering intensity as a function of the laser linear polarization angle. The dashed white line corresponds to a sinusoidal fit. (e) Visibility of SCO NPs as a function of their size at 642, 785, and 852 nm laser wavelengths. The solid lines were calculated by using Mie theory. The horizontal lines illustrate the experimental visibilities extracted from the data. The markers display intersections with Mie visibilities. The dashed vertical line corresponds to a size of 315 nm at zero laser excitation and ambient pressure. (f) Graph of the visibility at 642 nm versus NP size. These data were measured for three independent trapped SCO NPs irradiated at several laser intensities, as indicated by the color code on the right-hand side. (g) Relative laser-induced size change of trapped SCO NPs as a function of laser excitation intensity at λ = 488 nm. The relative sizes were retrieved following the method illustrated in panel (e). Red circles (P↑) correspond to the up-sweep, and blue triangles (P↓) to the down-sweep; dashed lines are guides to the eye. The vertical dashed line marks 6 mW, the power at which independent Raman measurements on non-trapped NPs indicate the HS state (Figure S4).

Using the trap and in situ microscope shown in Figure 2(a), we collect scattering snapshots at polarization angles of the probe laser beam ranging from 0 to 2π. These data provide the scattering response of isolated SCO NPs and, from it, their size under several laser excitation or pressure conditions. Figure 3(b),(c) presents a typical scattering microscopy snapshot of a levitating SCO NP and its corresponding integrated signal, where we fit a Lorentzian function to extract a background-free signal.

Figure 3(d) presents an example of the scattering response of an isolated SCO NP as a function of the laser polarization angle at 642 nm. We measure scattering curves at three wavelengths (642, 785, and 852 nm) to unambiguously extract the particle size. From the scattering curves, we extract optical visibilities by fitting sine functions to the data, which we then use to infer the particle size. The relationship between the electric field of the probe laser *E*_in_ and the scattered field *E*_sc_ depends on the dielectric function, size, and shape of the particle and on the laser wavelength λ and is given by

1 $$\left(\begin{array}{l} E_{∥}^{\mathrm{sc}}\\ E_{⊥}^{\mathrm{sc}} \end{array}\right)=\frac{\lambda e^{i2\pi r/\lambda }}{-i2\pi r}\left(\begin{array}{ll} S_{2} & 0\\ 0 & S_{1} \end{array}\right)\left(\begin{array}{l} E_{∥}^{\mathrm{in}}\\ E_{⊥}^{\mathrm{in}} \end{array}\right)$$

where *r* is the radial direction (with its origin at the center of the particle) and subscripts ∥ and ⊥ indicate the parallel and perpendicular components of the field with respect to the scattering plane, respectively. Equation 1 dictates that the elements of the scattering amplitude matrix |*S*_2_|^2^ and |*S*_1_|^2^ are proportional to the intensity scattered when the laser beam is fully polarized along the parallel or perpendicular direction, respectively (i.e., when *I*_∥_^in^ = *I*_0_ or *I*_⊥_^in^ = *I*_0_). These matrix elements describe the visibility as $V=\left({\left|S_{2}\right|}^{2}-{\left|S_{1}\right|}^{2}\right)/\left({\left|S_{1}\right|}^{2}+{\left|S_{2}\right|}^{2}\right)$ . The sign of visibility indicates which orthonormal component of the electric field leads to a maximum or a minimum of the scattered intensity.

We computed the elements of the scattering matrix and the visibility using the Mie formalism and the complex refractive index of the SCO NPs (see ellipsometry characterization in the Supporting Information). Figure 3(e) illustrates the calculated visibility as a function of the SCO NP size for three laser wavelengths with a period of λ/2 and an amplitude that strongly depends on the refractive index. The visibilities obtained from the experiments correspond to the horizontal lines in Figure 3(e), and the intersections with numerical calculations yield nine possible radii smaller than 1 μm. Thus, we employ three laser wavelengths to uniquely determine the size of the trapped particles. We allocate a Gaussian distribution centered at each intersection and calculate the convolution integral of the closest distributions at three wavelengths, providing a particle size of 315 nm (see the vertical line in Figure 3(e)). This value sets the initial particle size at ambient pressure in the absence of laser excitation, which, combined with new scattering experiments at 642 nm, is used to infer the subsequent particle’s size change for increasing laser excitation intensities. Figure 3(f) presents the visibility calculation at 642 nm (dashed-red line) along with experimental values (markers) obtained upon alternating laser excitation at increasing and decreasing laser powers. Overall, an increase in laser power leads to a higher temperature for the particle, which in turn induces a spin transition and a decrease in the acquired scattering visibility. Here, the laser-induced spin transition from the initial low- to the high-spin state of laser-heated particles leads to an increase in size.

Figure 3(g) provides the relative particle size change as a function of the laser excitation power, performed by cycling the laser power up and down. The analysis of these size–power curves reveals slightly asymmetric yet reproducible excitation-size trajectories across different NPs. The relative sizes are calculated by dividing the particle sizes at specific laser powers by the initial size in the absence of laser excitation. Our data reveal that increased excitation power induces a larger visibility shift (Figure 3(f)) and a larger particle size modification (Figure 3(g)). The increase from a normalized relative size of 1.00 to the observed maximum of ≈1.06 in Figure 3(g) leads to a laser-induced expansion of approximately ≈5 ± 1% (corresponding to a volume increase of Δ*V* ≈ 10%, while considering a rotational ellipsoid-shaped particle).

This abrupt light-induced size increase is consistent with typical values measured in SCO composite samples.^19,53–55^ Ellipsometry measurements (Figure S5c) further support this interpretation, showing negligible thermally induced thickness variations both above and below the transition temperature range. This observation is supported by independent Raman spectroscopy measurements on non-trapped SCO NPs, which identify the HS state at 6 mW (dashed vertical line in Figure 3(g); see Raman measurements in Figure S4). This power range also aligns well with the thermal bistability of the Fe­(II)–triazole coordination polymer and confirms the robustness of the spin transition under contact-free optical excitation.

### Pressure Modulation Experiments on Trapped SCO NPs

Next, we examine how decreasing the ambient pressure affects levitating SCO particles. Figure 4(a) shows representative 642 nm polarization scans as the pressure is decreased from 1013 to 0.1 mbar. The visibility V is positive at ambient pressure, flattens near ∼10^2^ mbar, and becomes negative at lower pressures, with sinusoidal amplitude recovering as pressure approaches a few mbar. The sign change and the evolution of the amplitude depend on the initial particle size and refractive index, consistent with the Mie calculations (Figure 3(e)–(f)). We tracked V(P) for six particles (Figure 4(b)) with an initial average NP size at ambient pressure of 280 ± 4 nm. The control particles introduced in the HS state (Figure 4(b1)) show pressure-insensitive visibilities, while the particles starting in the LS state exhibit a monotonic decrease of V that plateaus below ∼10 mbar (Figure 4(b2)).

**Figure 4:**
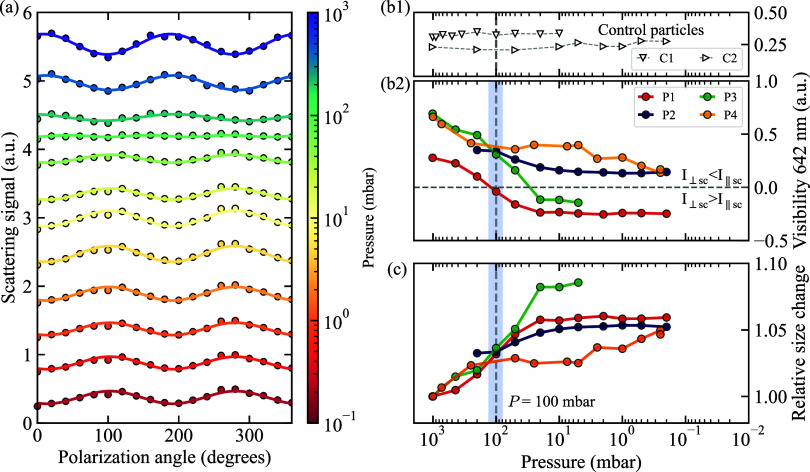
Pressure-dependent scattering of levitating SCO nanoparticles. (a) Experimental polarization-resolved scattering at λ_probe_ = 642 nm for a single trapped Fe­(NH_2_trz)_3_(NO_3_)_2_ nanoparticle, while the ambient pressure is reduced from 1013 to 0.1 mbar. Curves are vertically offset for clarity and colored according to pressure (log scale). (b) Experimental visibility *V* at 642 nm for six isolated particles as a function of ambient pressure. Their initial average NP size at ambient pressure is 280 ± 4 nm. (b1) Control particles (C1 and C2) introduced in the high-spin state exhibit pressure-independent *V*. (b2) Particles initially in the low-spin state show a monotonic decrease in *V* that saturates below ∼10 mbar. The vertical dashed line marks the approximate crossover pressure, and the horizontal dashed line separates *I*_Lsc_ < *I*_∥sc_ (*V* > 0) from *I*_Lsc_ > *I*_∥sc_ (*V* < 0). (c) Relative particle size change, extracted using the multiwavelength visibility method, as a function of ambient pressure.

Using the multiwavelength visibility method (Figure 3(e)–(f)), we converted these data to relative size changes (Figure 4(c)). Reducing the pressure from ambient pressure to 10^–1^ mbar produces a systematic decrease and ultimately a sign change in visibility, together with a 4–6% increase in the inferred particle size. The most plausible origin of this “vacuum-induced” dilatation is dehydration of the Fe­(II)–triazole coordination polymer: under vacuum, the particles lose both weakly physisorbed water at the surface and a fraction of lattice (structural) water. Removal of these molecules (i) releases surface-induced compressive stress and capillary forces that clamp the lattice and (ii) weakens the hydrogen-bonding network that links NH_2_ groups and NO_3_^–^ anions to the triazole chains. The latter reduces the effective ligand-field splitting at Fe­(II), tipping the LS → HS balance toward the expanded HS geometry and leading to the observed volumetric increase via elastic cooperativity. Notably, because dehydration can modify local crystal packing and hydrogen-bond topology, this mechanism may impart a partial or even irreversible bias toward the HS state, which is not necessarily recovered upon subsequent repressurization cycles. Note that pressure acts indirectly by promoting dehydration of the nanoparticles, and is therefore not interpreted as a direct thermodynamic control parameter of the spin transition. The visibility “turning point” near ∼10^2^ mbar is consistent with a threshold at which the desorption rate of water/solvent exceeds readsorption; it also aligns with observations on related Fe–triazole frameworks where depressurization into the 10^2^ mbar regime biases the equilibrium toward HS.^56^ Secondary contributions—reduced gas-phase cooling and damping at low pressure, which allow modest optical dissipation to raise the steady-state temperature—could potentially assist the shift but are insufficient to account for the magnitude on their own. The control particles prepared in the HS state show pressure-insensitive visibilities (Figure 4(b1)), and the purely thermal expansion expected within a fixed spin state is far below the measured relative size increase of 5 ± 1%, both observations reinforcing a dehydration-driven LS → HS conversion. The particle-to-particle spread in the apparent crossover pressure (Figure 4(b2)–(c)) is readily ascribed to variations in size, defect density/tilt boundaries, initial water loading, and net charge, which modulate both desorption kinetics and local spin-state equilibrium.

## Conclusions

We establish a substrate-free platform for manipulating and interrogating spin crossover at the single-NP level. By coupling a quadrupole Paul trap with a multispectral, polarization-resolved scattering microscope, we isolate individual Fe­(NH_2_trz)_3_(NO_3_)_2_ nanoparticles, excite them optically, and read out their state in real time while independently tuning the surrounding pressure. This configuration provides three key capabilities that have been challenging to achieve: (i) contact-free confinement that preserves the intrinsic response of the nanoparticle, (ii) quantitative three-dimensional metrology of size and refractive-index-dependent scattering, and (iii) precise environmental control (laser intensity and pressure) at the level of a single particle.

Using this platform, we resolve laser-induced thermally driven switching between spin states with reversible optovolumetric expansions up to ∼10% and well-defined power thresholds. Independent pressure modulation yields a comparable expansion, and control measurements on particles prepared in the HS state remain insensitive to the pressure. We attribute the observed “vacuum-induced” dilatation to dehydration of the Fe­(II)–triazole coordination polymer, which can reduce the effective ligand-field splitting in Fe­(II), shifting the LS ↔ HS equilibrium toward the expanded lattice that produces the observed volumetric increase. Importantly, the effects of reduced pressure are mediated by dehydration and may not be fully reversible upon repressurization.

Besides providing a clear, mechanistic picture of single-particle switching, these results define operational windows, such as intensity, pressure, and temperature, for integrating SCO NPs into nanophotonic circuitry. The ability to deterministically toggle and read out the spin state of an individual particle, free from substrate artifacts, supports energy-efficient optical switches, nonvolatile elements for dense data storage, and nanoscale sensors whose transduction rests on large, reversible volumetric and refractive index changes.

We present a general methodology that can be extended to other SCO materials and molecular phase-change systems, to smaller-sized particle, and to architectures that couple levitated particles to optical cavities or integrated resonators for enhanced readout and feedback control. Combining the present approach with ultrafast excitation and on-chip trapping will enable time-resolved studies of cooperative dynamics, defect motion, and fatigue at the single-crystal level. Together, our work provides guidance on the design and deployment of molecular phase-change building blocks for reconfigurable, ultralow-power nanophotonics.

## Experimental Methods

### SCO Nanoparticle Synthesis and Characterization

#### Synthetic Protocols

All chemical reagents, including tetraethyl orthosilicate 98% (Sigma-Aldrich), Triton X-100 (Sigma-Aldrich), ascorbic acid (Sigma-Aldrich), 4-amino-4*H*-1,2,4-triazole (Sigma-Aldrich), iron tetrafluoroborate hexahydrate (Sigma-Aldrich), barium nitrate (Sigma-Aldrich), Silicon Elastomer Sylgard 18 kit (Sigma-Aldrich), polymethyl metacrylate (Mw 35,000, Sigma-Aldrich), n-hexanol (Sigma-Aldrich), cyclohexane (Sigma-Aldrich), ultrapure water (18.2 MΩ), absolute ethanol (extra dry, 99.5%, AcroSeal) and HPLC-grade acetone (Scharlau), were purchased and used without further purification.

We synthesized Fe­(NH_2_trz)_3_(NO_3_)_2_ SCO NPs following the reverse micelle protocol, which consists of blending two separate microemulsions of the metal, Fe^2+^, and the ligand 1,2,4-amino triazole. This protocol enabled us to control the size of the produced nano-objects by fine-tuning key reaction parameters.^50^ In the IR spectra, we observed the characteristic stretching vibrations of the amine-triazole group at around 1500 cm^–1^ for C=C and N=N from the triazole and in the 3100–3300 cm^–1^ range corresponding to NH_2_ and N–H groups of the amine (Figure SX). XRD analysis showed that the material is highly crystalline and consists of a single phase, with the pattern matching the theoretical LS pattern well. NP size distributions in solution were determined in ethanol (0.1 mg/mL) suspensions by DLS using a Zetasizer ZS (Malvern Instrument, U.K.). Transmission electron microscopy studies were carried out on a Tecnai G2 F20 microscope operating at 200 kV. Samples were prepared by dropping suspensions on lacey Formvar/carbon copper grids (300 mesh). The real size distribution was determined by user-assisted counting of TEM images by using ImageJ software. Attenuated total reflectance Fourier transform infrared spectra were collected using an Alpha II FTIR spectrometer (Bruker) in the 4000–400 cm^–1^ range without KBr pellets. Powder X-ray diffraction measurements were carried out using a PANalytical Empyrean diffractometer employing Cu Kα radiation (Cu Kα = 1.5418 × 10^–10^m) with a PIXcel detector, operating at 40 mA and 45 kV. Profiles were collected in 2° < 2θ < 45° range with a step size of 0.013°. UV–vis absorption spectra were recorded on a Jasco V-670 spectrophotometer in baseline mode from 400 to 800 nm using Thermo Scientific 96-well UV microplates. Magnetic data were collected with a Quantum Design MPMS XL-5 susceptometer equipped with a SQUID sensor. DC FC magnetization measurements were performed under an applied magnetic field of 100 Oe at a scan rate of 1 K min^–1^ in the temperature range from 100 to 400 K.

### Trap and Electrospray Systems

The trap setup combines an electrospray system, a vacuum chamber, and a linear Paul trap. The Paul trap is built using four parallel gold-coated rods arranged to form a square of side *L* = 8 mm (*L*_rods_ = 12 cm and *r*_rods_ = 3 mm). The shape and size of the rods provide optical access to trapped particles, while the so-produced electric field can be approximated to a field generated using hyperbolic rods.^41,57,58^ Rods placed at diagonally opposite vertices are attached to an amplifier that provides an AC voltage (*U*_pp_ = 600 V and Ω = 3.0 kHz), while the other pair of rods is connected to a DC voltage (*U* = 0–5 V). In this configuration, our setup generates a time-varying electric quadrupole potential that can trap single nano- to micron-sized particles, following the so-called Mathieu equations.^37^ The SCO particles are introduced in between the rods of the trap by means of an electrospray system, which employs a syringe with a metallic needle that is set to 2900 V. Such an arrangement is placed close to the grounded trap entrance plate, thus originating a spray of charged particles with a conical shape (i.e., Taylor cone). The syringe contains a mixture of approximately 20% SCO particles and 80% spectrally pure ethanol. SCO experiments were performed on a variety of particles across different experimental runs and days, which can lead to slightly different charging conditions. Nevertheless, our measurements yielded consistent results with no indication that, in this context, charge-related effects significantly influence the SCO response. The aforementioned time-dependent quadrupole electric potential allows our trap to effectively act as a charge-to-mass ratio (*q*/*m*) selector, where the electric force that balances gravity scales with *q*/*m*, and particles are trapped, provided their *q*/*m* ratio lies within the Mathieu-stability region, which offers practical leeway (i.e., modest variations in charge do not compromise stable confinement). Once the trapped particles are stable, we lock their positions along the trap axis by activating the segmented rods. Subsequently, we activate the vacuum pump to gradually extract the air inside the transparent vacuum chamber.

### Polarization-Resolved Scattering Microscopy

The automated microscopy and polarimetry systems allow us to record snapshots of the scattered laser light intensity from levitated particles as a function of the laser polarization angle, as shown in Figure 3(a). The polarimetry system uses a multiplexer to combine three linearly polarized pigtailed diode lasers with wavelengths 642, 785, and 852 nm. We employ these lasers to illuminate the trapped particles in order to acquire their scattering response and use it to characterize their size.^41^ During the automated experiments, the laser polarization angle is set using an achromatic lambda half-wave plate (350–850 nm, Thorlabs) mounted on a motorized rotation stage (PRM1/MZ8, Thorlabs). For each polarization angle, we collect 5–10 scattering micrographs using the in situ microscope. The microscope employs a tube lens (*f* = 50 mm) and a CCD camera (Zelux, Thorlabs) to record images of the scattering originating from the illuminated trapped particles at a 90° angle with respect to the laser propagation direction. Each image is integrated along the *y*-axis and fitted using a Lorentzian function to extract a background-free scattering signal, as shown in Figure 3(b). For each polarization angle, we average the scattering signal provided by the set of snapshots, yielding the scattering as a function of the laser probe (642 nm) polarization angle under different laser excitation conditions (488 nm). Although our SCO NPs are elongated, their rotational motion in the trap leads to an orientation-averaged optical response; therefore, the Mie analysis yields an effective spherical optical radius. Figure 2(c) presents an example of such curves for a trapped particle at ambient pressure (1013 mbar). In this case, the probe laser emits at λ_p_ = 642 nm. However, we measure the scattering signal at three wavelengths of 642, 785, and 852 nm in order to unambiguously extract the particle size. To this end, sinusoidal fits to the polarization-resolved scattering signal aid in extracting the visibility at three wavelengths, which is then used to infer the particle size as we explain in the following.

We run two types of experiments to inspect the scattering response of trapped particles as a function of pressure and moderate laser pump intensities. For the first experiments, trapped particles are investigated at specific pressure values inside the vacuum chamber in the 0.01–1013 mbar range. To this end, we use a coarse-and-fine valve system (Edwards) that connects the vacuum chamber of the trap to a scroll pump (Edwards). The pressure inside the chamber is monitored using a gauge (TPG50 and TPG51, Balzers). In order to prevent particles from exiting the trap during subsequent air exhaust procedures, we employed trap segments to generate an additional electric potential that retains the particles along the trap axis. For the laser excitation experiments, we employ a CW blue pump laser at 488 nm (LP400-SF20G), which delivers powers ranging from 30 μW to 100 mW. The laser diode is embedded in a temperature- and impedance-controlled mount (LDM9LP, Thorlabs) connected to a power supply (ITC4000, Thorlabs). The laser is guided via fiber optics to a port, where a collimator directs the beam to a microscope objective (Mitutoyo, Plan-Apochromat objective, 10×, NA = 0.28, working distance 34 mm). The laser fiber and objective lens are mounted on a three-dimensional translation stage that allows us to optimize the beam-particle alignment in real time by maximizing the scattered light collected by the microscope. Details of the laser beam characterization procedure are provided in the Supporting Information. To estimate the precise laser fluence illuminating the SCO nanoparticle surface, we use the formula that accounts for the energy per particle, as detailed elsewhere.^59,60^ We filter out the laser excitation wavelength employing a long-pass filter (FGL530M, Thorlabs) in order to acquire 405 nm-free snapshots during the polarimetry experiments.

## Supplementary Material





## Notes

Video illustrating color change of SCO nanoparticles (MP4)
Extended details of the experimental methods and laser beam diagnostics; IR, PXRD, ζ-potential, Raman spectroscopy, absorption, and DLS characterization of the SCO nanoparticles; and characterization of the complex refractive index of SCO particles using ellipsometry (PDF)
