# Supplementary material for: Unveiling Spin Transition at Single-Particle Level in Levitating Spin Crossover Nanoparticles
Source: ACS Nano. 2026 Feb 3;20(6):5044–53. doi: 10.1021/acsnano.5c18794 (PMC12918722; doi:10.1021/acsnano.5c18794)
Supplement: Supplementary file 2 [file nn5c18794_si_002.pdf]

Unveiling Spin Transition at Single Particle Level in Levitating Spin Crossover Nanoparticles: Supporting Information

Elena Pinilla-Cienfuegos,*,† Lucas Mascaró-Burguera,† Ramón Torres-Cavanillas,‡ J. Ignacio Echavarría,¶ Alejandro Regueiro,‡ Eugenio Coronado,‡ and Javier Hernandez-Rueda*,¶

†*Nanophotonics Technology Center, Universitat Politècnica de València, 46022 Valencia, Spain*

‡*Instituto de Ciencia Molecular, Universitat de València, 46980 Valencia Spain*

¶*Department of Optics, Faculty of Physics, University Complutense of Madrid. Plaza de Ciencias 1, 28040 Madrid, Spain*

E-mail: epinilla@ntc.upv.es; fj.hernandez.rueda@ucm.es

S1. Zeta-potential measurements

Zeta-potential characterization of $[Fe(NH_2trz)_3](NO_3)_2$ NPs

To confirm the presence of surface charges in isolated SCO nanoparticles, we performed ζ-potential measurements (Fig. S1). The nanoparticles exhibit a ζ-potential of approximately -4 mV, consistent with the partial surface charging expected from the presence of multiple NO_3^- counterions in the structure. This observation supports the suitability of $[Fe(NH_2trz)_3](NO_3)_2$ as a platform for the preparation of voltage-responsive SCO nanomaterials.

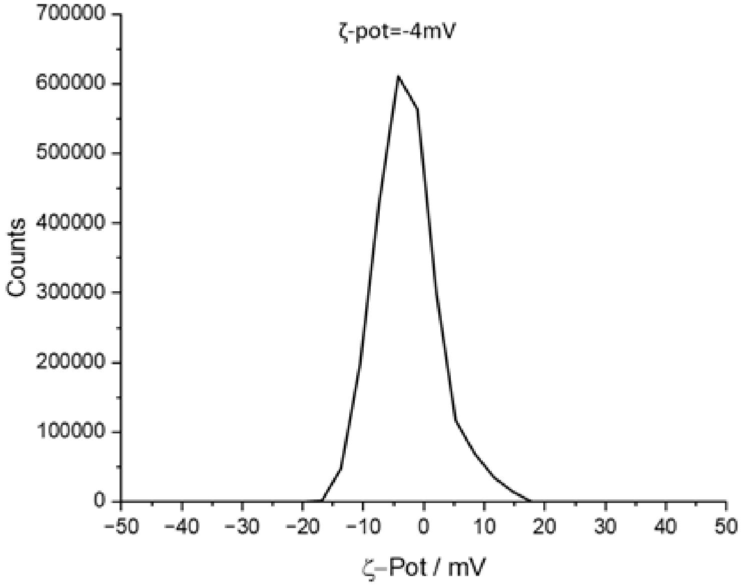

Figure S1: ζ-potential measurements.

S2. Dynamic Light Scattering measurements

In Fig. S2, we plot a dynamic light scattering (DLS) measurement of our [Fe(NH_2trz)3(NO_3)$_2$] nanoparticles. The measurements were performed at room temperature using a Zetasizer ZS (Malvern Instrument, UK). The size distribution curve reveals a well-defined peak centered around 200 nm, indicating distinct populations of nanoparticle as small as 130 nm and as large as 400 nm.

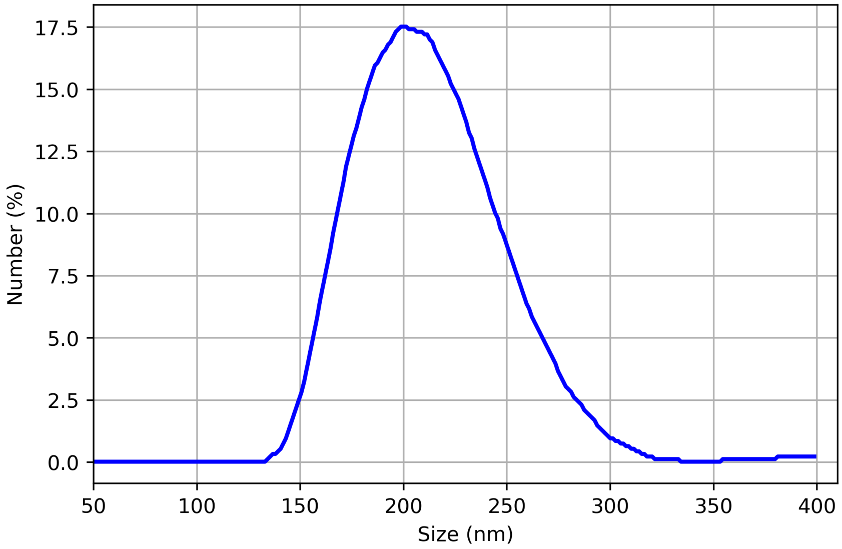

Figure S2: Dynamic Light Scattering measurements

S3. Infrared and X-Ray Diffraction measurements

In Fig. S3 we illustrate (a) infrared (IR) spectra and (b) powder X-ray diffraction (PXRD) pattern of the Fe(II)–triazole coordination polymer before (MMC, black) and after (AGR, blue) the nanoparticle fabrication process. The IR spectra confirm the preservation of the characteristic vibrational modes of the triazole-based framework, with only minor shifts observed, indicating retention of the molecular structure. The PXRD pattern exhibits sharp and well-defined diffraction peaks, consistent with a crystalline structure, and in agreement with previously reported patterns for this compound class. These results validate the chemical integrity and crystallinity of the SCO material throughout the fabrication and processing steps.

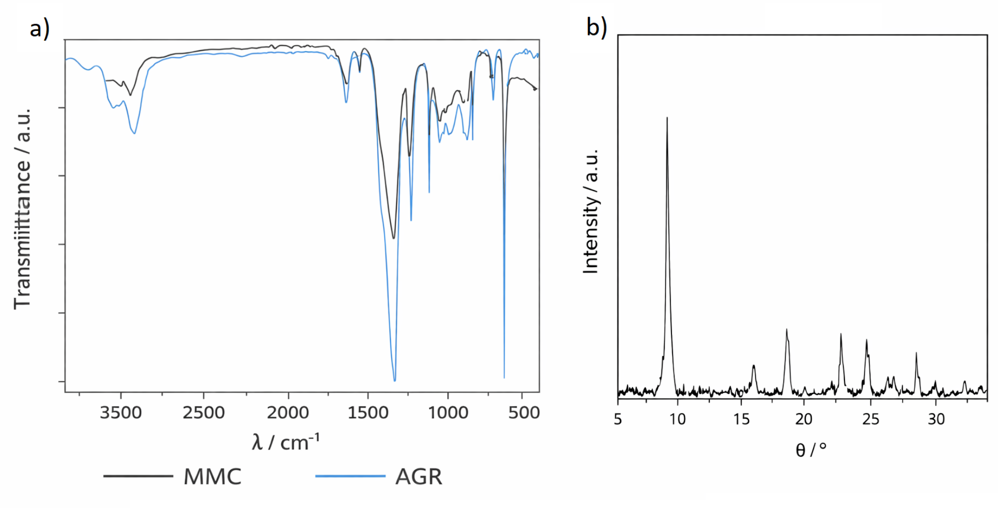

Figure S3: a) Infrared and b) Powder X-Ray Diffraction measurements

S4. Measured Raman spectra

Raman spectroscopic characterization was carried out using an HR Evolution confocal Raman microscope (Horiba) with a laser spot size of approximately 1 μm (Olympus LMPlanFl 50x LWD, NA 0.50) in backscattering geometry. A laser with an excitation wavelength of λ_{exc}= 473 nm was used to induce the spin state transition, while λ_{exc}= 633 nm served as the probe for spin state detection. The incident laser power for both lasers was maintained at 25% of the maximum which corresponds to 6 mW for the blue laser (473 nm). A diffraction grating with 600 grooves/mm was used.

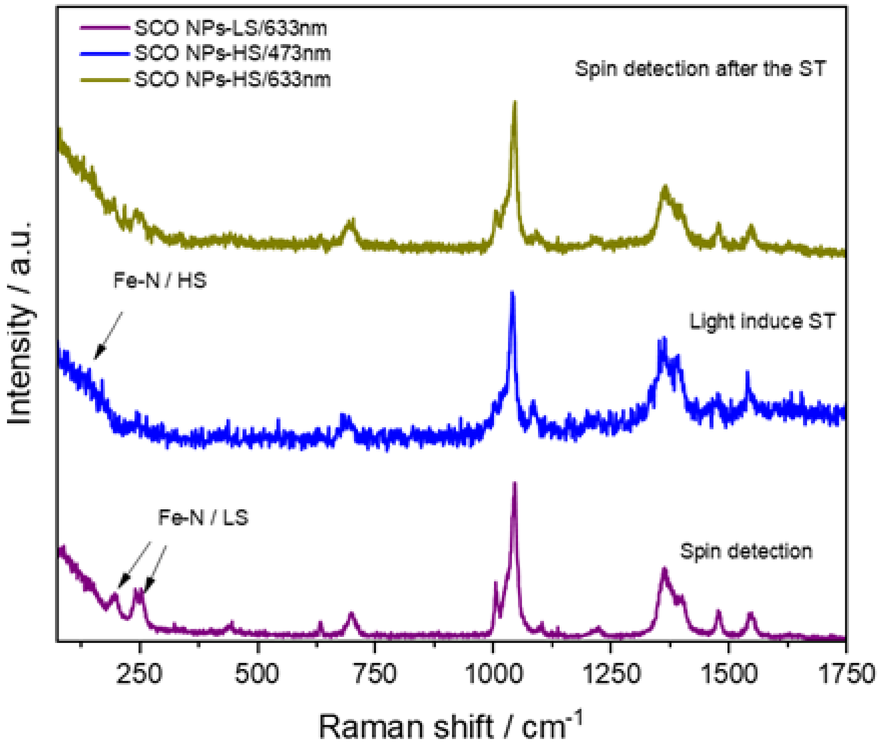

Figure S4: Raman spectra of the SCO NPs measured with a 633 nm excitation wavelength (bottom). Spectra recorded with a 473 nm excitation wavelength for the light-induced spin transition (middle). Finally, the vibrational signature of the NPs after the spin transition, measured again with a 633 nm excitation wavelength (top).

S5. Elipsometry measurements

Ellipsometric analysis of the nanocomposite thin film. The sample, consisting of SCO/PMMA, was spin-coated onto a silicon substrate, yielding a uniform and optically smooth film. The film was characterized with a UV-visible-NIR SENTEC spectroscopic ellipsometer equipped with a programmable heating stage (from 300 K to 350 K). Spectroscopic ellipsometry enabled us to determine the complex refractive index ($n + ik$) of the $Fe(NH_2trz)_3(NO_3)_2$ compound embedded in the polymer matrix.

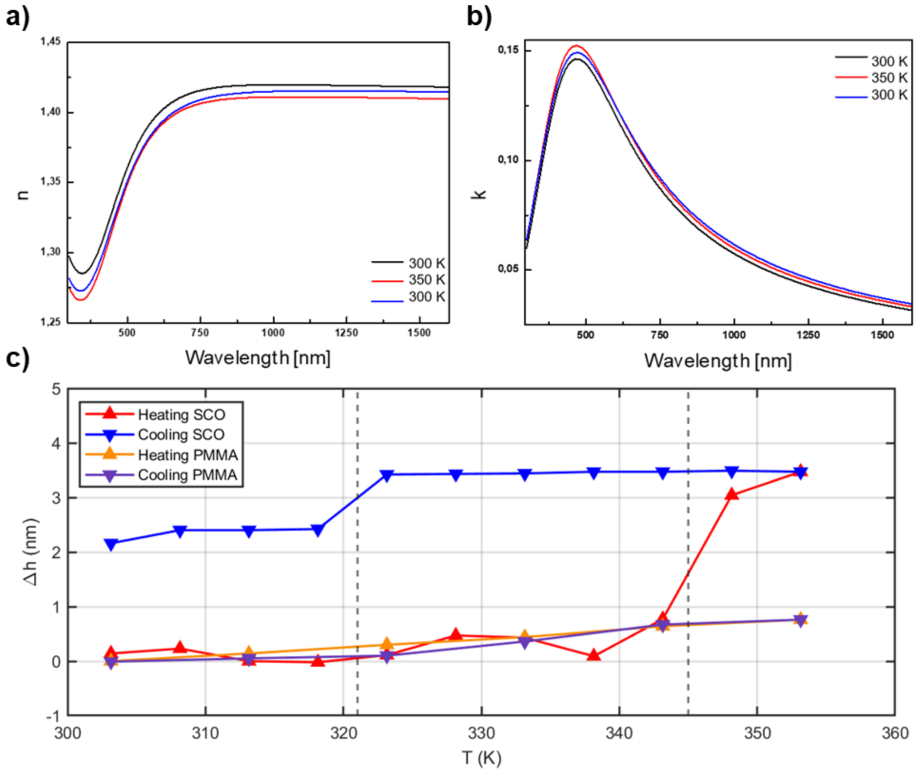

Figure S5: Ellipsometry data. (a) Refractive index (n) and (b) extinction coefficient (k) as a function of wavelength at different temperatures (300 K, 350 K, and 300 K after cooling). (c) Temperature-dependent thickness variation (Δh) during heating and cooling cycles for SCO/PMMA and PMMA films.

The results from the SCO plus PMMA resist layer were fitted from ellipsometry data using Tauc–Lorentz oscillators,[1] allowing an accurate description of both the dispersion of the refractive index (n, Figure 5a) and the extinction coefficient (k, Figure 5b), as a function of wavelength at different temperatures (300 K, 350 K, 300 K cooling). Figure 5c shows

the temperature-dependent thickness variation (Δh) of the SCO/PMMA and PMMA films. For the SCO/PMMA layer, thermal hysteresis is observed for the SCO/PMMA layer, where the PMMA reference displays a linear increase in thickness (below 1 nm) throughout the temperature range.

S6. UV–Vis absorption spectrum at HS configuration

After redispersing the SCO NPs in ethanol, we heated the dispersion to 60 °C to induce the spin transition and stabilize the high-spin (HS) configuration. The resulting UV–Vis absorption spectrum (Figure S6) confirms the HS state, as the broad band centered at 520 nm ($\Delta\lambda \approx 100$ nm) characteristic of the low-spin (LS) state is no longer observed. Consistent with this spectral change, the dispersion loses its typical LS pink coloration and becomes milky-white in the HS state. Upon subsequent mechanical agitation of the vial, a gradual recovery of the pink coloration is observed (Supporting Movie), indicating a HS→LS back-transition. This effect is attributed to transient cooling of the dispersion caused by heat exchange with the colder vial and the surrounding environment at room temperature, in line with the thermally driven and reversible nature of the spin crossover near ambient conditions.

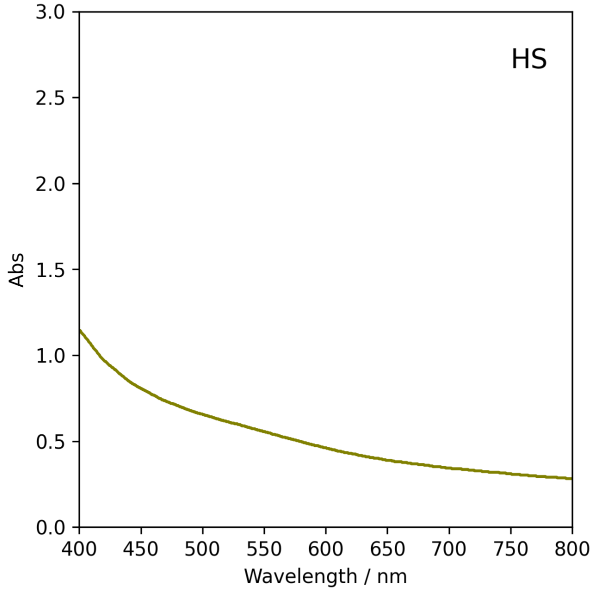

Figure S6: UV-Vis absorption spectrum of SCO nanoparticles in ethanol after heating to $60\,^{o}$C to induce the LS-to-HS transition, showing disappearance of the LS band around 520 nm.

S7. Pump laser beam size characterization inside the trap

The calibration of the size of the pump laser beam that was focused on the trapped particles in the trap was carried out at a wavelength of 488 nm. The focused laser beam was scanned laterally across a static trapped particle, and the corresponding scattering signal was recorded as a function of the beam position along the x-axis parallel to the trap axis. The experimental profiles were analyzed by fitting a Gaussian function to reliably determine the spatial beam distribution. This procedure was repeated multiple times to ensure reproducibility and reduce statistical uncertainty.[2]

References

(1) Jellison, G. E.; Modine, F. A. Parameterization of the optical functions of amorphous materials in the interband region. *Applied Physics Letters* **1996**, *69*, 371–373.

(2) Maestro, L. M.; Antón, M. A.; Cabrera-Granado, E.; Weigand, R.; Hernandez-Rueda, J. Intrinsic Optical Response of Levitating Upconverting Single Particles. *ACS Photonics* **2025**, *12*, 1783–1792.
